# Electromyography of the stapedius muscle via a retrofacial approach and electrically evoked stapedius reflex during cochlear implant surgery: a prospective bicentric study

**DOI:** 10.1038/s41598-026-53093-3

**Published:** 2026-05-13

**Authors:** Orlando Guntinas-Lichius, Dirk Arnold, Gerd Fabian Volk, Daniela Korth, Daniel Richter, Rene Aschenbach, Fritz Schneider, Thore Schade-Mann, Philipp Gamerdinger, Jose Luis Vargas Luna, Anke Tropitzsch, Hubert Löwenheim

**Affiliations:** 1 Department of Otorhinolaryngology, Facial-Nerve-Center, Jena University Hospital, Jena, Germany; 2 Department of Radiology, Jena University Hospital, Jena, Germany; 3 Department of Otolaryngology-Head & Neck Surgery, Hearing Research Center, University of Tübingen Medical Center, Tübingen, Germany; 4 Department of Otolaryngology and Communication Enhancement, Boston Children’s Hospital, Boston, MA US; 5 Manton Center for Orphan Disease Research, Boston Children’s Hospital, Boston, MA US; 6 Department of Otolaryngology, Harvard Medical School, Boston, MA US; 7 MED-EL Medical Electronics, Innsbruck, Austria; 8 Department of Otorhinolaryngology, Jena University Hospital, Am Klinikum 1, D-07747 Jena, Germany

**Keywords:** Cochlear Implantation, Electrical Stimulation, Reflex, Stapedius Reflex, Electromyography, Engineering, Health care, Medical research, Neurology, Neuroscience

## Abstract

Measuring the electrically evoked stapedius reflex threshold (eSRT) with electromyography (EMG) of the stapedius muscle (SM) may offer an objective approach for cochlear implant (CI) fitting. During unilateral CI surgery in 14 adult patients with single-sided deafness, a retrofacial or anterior approach were used to evaluate the feasibility of SM-EMG during acoustically and electrically elicited stapedius reflex (SR). No adverse events occurred. This neurophysiology-based method was compared with visual detection of the SR. Intraoperatively, the belly of the SM was accessed via the retrofacial approach in 11 patients and via an anterior approach in 3 patients. Objective SR determination using electrical stimulation through the CI combined with SM-EMG was feasible in 7 patients using the retrofacial approach, but in only 1 patient using the anterior approach. Overall, electrical stimulation elicited SM-EMG responses in 8 patients, whereas acoustic stimulation elicited responses in only 3 patients. When eSRT measurement via SM-EMG was possible, stimulation thresholds were equal or slightly lower than thresholds identified visually in 85% of all SR measurements. SM-EMG-based eSRT appears to be a viable approach to estimate the eSRT intraoperatively in CI users as it shows similar performance as current clinical intraoperative standard. Further investigation is needed to investigate the relationship between intraoperative SM-EMG-based eSRT and patients’ comfort levels, establish correction factors, and potentially enable integration into future closed-loop smart CI devices.

## Introduction

Estimates indicate around 30.7 million people have severe hearing loss, with another 17.2 million having profound hearing loss^1^. A cochlear implant (CI) is an auditory prosthesis that allows rehabilitation of hearing to people affected with severe or profound hearing loss via electrical stimulation of the acoustic nerve. For the CI to work effectively, stimulation parameters must be set-up in so called fitting sessions.

Nowadays, the main focus when fitting a CI lies in setting the maximum and minimum intensity levels per electrode (definition of the dynamic range)^2^. In standard clinical practice, these levels are mainly based on the CI users’ subjective perception of loudness and the users’ pure tone and speech audiometry; objective measures are used to a lesser extent^2^. The accuracy of fitting the CI based on subjective perception is impacted inevitably by the CI users capacity to reliably complete the associated tasks, bearing in mind their developmental and cognitive ability^3^. Thus, to provide more accurate fitting the use of objective measures would be considered desirable. Of the objective measures available, levels determination based on electrically evoked compound action potentials (eCAP) are used in 85% of cases; while fitting based on the electrically evoked stapedius reflex threshold (eSRT) is used in up to 39% of centers^2^.

The eSRT measurement has significant potential as an objective measure in CI fitting. It relies on the stapedius reflex (SR), an autonomic reflex that protects the inner ear from noises above the safe threshold. During the reflex the stapedius muscle (SM) contracts. The SM is connected to the stapes by a tendon and the muscle contraction increases the impedance of the ossicular chain. The SR can be triggered acoustically by noises above a certain sound pressure level^4^, and in CI users in a controlled clinical environment, the SR can be triggered electrically by the CI electrode within the cochlea. The amount of electrical stimulation needed to elicit the initial SR is referred to as the threshold level. Several studies indicate that eSRTs are closely correlated with the most comfortable loudness (MCL) levels in children and adults^5–11^. Thus, eSRT is an appropriate measure in the definition of the patient’s dynamic range during CI fitting.

The clinical standard for SR assessment is the non-invasive detection in the outer ear canal of the related admittance-induced changes during the reflex. Such a procedure is however an indirect measure of the SM activity^12^. Furthermore, it rules out measurements under conditions where the biomechanics of the ear are not intact e.g. a missing stapedius tendon^13^, or in cases with middle-ear malformation. Thus, a direct objective measure of the SM activation and the related recording of eSRT is of great interest. In fact, it could improve CI fitting of children and patients unable to provide the subjective/behavioral feedback currently needed to fit a CI post-surgery. Investigations on safe and reproducible methodologies and procedures for permanent measure of the eSRT are also very appealing since they could drive the development of future closed-loop smart CI devices and remote fitting procedures.

Since the SM is encased by bone with only a narrow opening for the tendon that connects it to the stapes we endeavored in our earlier work, to identify a safe and reproducible surgical procedure to directly access the SM. The retrofacial approach was established as the most suitable approach in a cadaveric model^14^. Subsequently, we were able to gain access to the SM via the retrofacial approach in humans during standard CI surgery^15,16^. This intraoperative setting was used as a proof‑of‑concept platform for direct SM‑EMG monitoring, rather than as a routine alternative to established objective measures such as eCAP or eABR.

In the present study, our primary aim was to determine the intraoperative feasibility of directly recording electromyographic (EMG) activity from stapedius muscle (SM) during elicitation of the stapedius reflex (SR). As a secondary goal, we evaluated whether SR thresholds derived from SM-EMG, obtained via either a retrofacial or anterior approach, were comparable to those determined using the current intraoperative standard, the surgeon’s subjective visual identification of stapedius tendon movement.

## Methods

### Study design

This study was an experimental, acute, prospective, transversal,, bicentric clinical trial. The study was registered at German Clinical Trials Register (DRKS00019939; https://drks.de/search/en/trial/DRKS00019939; date of registration: 03/03/2020). Data were collected from 2016 to 2022, at the Department of Otorhinolaryngology, Jena University Hospital, Germany, and at the Department of Otorhinolaryngology, University of Tübingen Medical Center, Tübingen, Germany. The study was conducted in accordance with the Declaration of Helsinki and approved by both local ethics committees (Ethics Committee of the Jena University Hospital, Jena, Germany, and Ethics Committee at the University Hospital of Tübingen, Tübingen, Germany). Patients gave written informed consent for inclusion in this study. Adult patients with (1) single side deafness with no residual hearing but normal/near-normal hearing on the contralateral side, (2) planned for CI surgery, 3)) who opted for a MED-EL CI, and 4)) with a measurable acoustic SR from the contralateral side were included.

### Pre-operative audiological screening

During screening, acoustically evoked SR were elicited and measured on both sides. Acoustic stimulation was performed at 4 different frequencies: 500, 1000, 2000 and 4000 Hz; and, at intensities between 80 and 100 dB during screening. Stimulation length was 300 ms each time, at intervals of at least 1000 ms. Standard commercial tympanometry devices were used (eTymp USB, BioMed, Jena, Germany, and Titan, Interacoustics, Middelfart, Denmark).

### Pre-surgical planning

In preparation for CI surgery DynaCT images of the middle ear of each patient were collected to evaluate the characteristics of the anatomy and classify the SM exposure according to criteria of Volk et al.^16^. The DynaCT images were then used to generate 3D reconstructions, as described by Marquez et al.^17^, for reference during surgery. In brief, segmentation of the DynaCT images were performed followed by a segmentation of all anatomical structures relevant to the surgery. Finally, based on the computed distances between structures, the surgical planning tool used automatized algorithms to generate a series of trajectories from the mastoid cavity to the SM and compare their feasibility and safety to reach the SM via a retrofacial approach^17^.

### Surgery

All surgeries were performed under general anesthesia. All study participants received a total intravenous anesthesia (TIVA) with remifentanil-propofol. Muscle relaxation with succinylcholine as a short-acting neuromuscular blocking agent was only used to induce a rapid, temporary muscle paralysis for intubation. The effect of succinylcholine lasts only 5–10 min. The surgical approach for CI was a standard mastoidectomy and posterior tympanotomy until the facial recess was exposed and the course of the facial nerve (FN) to the tendon of the SM was identified. The SM was accessed according to the classification of the anatomical configuration as determined during pre-surgical evaluation using the DynaCT 3D reconstructions^15,16^. During surgery the reconstructions were then visually aligned with the real position of the patients head to provide the surgeon a visual reference of the position of the FN and SM.

In the retrofacial approach, the surgical spot was drilled posterior and medial to the mastoid portion of the FN almost half-way between the identified level of the stapes head and the branching of the chorda tympani, as described in Guntinas-Lichius et al.^15^. During the whole surgery, a facial nerve monitoring device was used to control the position of the FN. If the retrofacial approach was not feasible in instances when the SM was completely covered by the mastoid portion of the facial nerve, an anterograde approach was performed, i.e., the SM was exposed along the stapedius tendon^22^.

### Intra-operative procedure

#### Contralateral acoustic stimulation (physiological check and visual/EMG assessment)

Acoustic reflexes on the contralateral side were first checked after anesthesia induction, but before the start of surgery. They were then measured again after mastoidectomy and before inserting the CI electrode, with an additional visual inspection performed on the operated side (tendon movement). These repeated assessments served as a physiological checkpoint to verify the stability of the reflex, monitor potential recording issues, and distinguish a true absence of response from technical problems and have not been considered for the data analysis.

After surgical access to the SM according to the procedure described in Guntinas-Lichius et al.^15^, the paired electrodes were inserted either through the retrofacial approach into the muscle belly, or via the anterior approach above the stapedius tendon when retrofacial insertion was not feasible. At this point, contralateral acoustic stimulation was performed once more. Reflex presence was assessed both visually and via EMG recording. Acoustic stimuli were presented at 500, 1000, 2000, and 4000 Hz, with intensities increased in 5 dB steps from 90 to 110 dB. The stimulation bursts were initially 500 ms and later reduced to 300 ms to follow CI optimization. The same tympanometry devices used pre-operatively (eTymp USB, BioMed, Germany; Titan, Interacoustics, Denmark) were also used intra-operatively.

#### Electrical stimulation via the CI (visual confirmation and EMG measurement)

Once the acoustic stimulation was done, EMG electrodes were temporarily removed and CI electrode array was inserted. Then, a standard intraoperative impedance telemetry (routine MED-EL procedure) was performed to verify electrode integrity and channel function. Throughout the study, several opportunities for optimizing EMG recordings during CI stimulation were identified. Accordingly, acquisition parameters and the type of needle electrodes were modified for the final eight patients (patient 7–14).

CI stimulation was controlled with the ESRT task in Maestro 6.0.1 or later (MED-EL, Innsbruck, Austria) using the MAX programming interface (MED-EL, Innsbruck, Austria) to connect the external coil that drives the CI system. Stimulation rate was set to either 2000 pulses per second (pps) or 250 pps, but kept the same for all channels within the same patient. Similarly, burst duration was initially set to 500 ms, and later reduced to 300 ms to reduce measuring time.

CI stimulation usually started with a medial electrode, e.g., contact 6, and proceed alternating between basal and apical contacts (e.g., contacts 6, 9, 3, 11, 1, etc.), allowing sampling across the array. Depending on surgical conditions, contact status, and available intraoperative time, either a single channel or multiple channels (up to all available contacts) were measured in an individual patient. For the first stimulated contact, the intensity sweep began at a very low intensity level typically 5 qu—*qu* or *charge unit* is equivalent to approximately 1 nCoulomb and is implemented in MED-EL CIs as intensity unit. For subsequent contacts, the starting intensity was adjusted based on the threshold (or maximum tested level) from the preceding contact to avoid unnecessary subthreshold stimulation, effectively reducing measurement time. Intensity was then varied in 6% steps of the prior value, with smaller changes applied near the expected threshold. SM-EMG responses were recorded, and each detected EMG response was repeated to confirm reproducibility. In parallel, the operating surgeon visually inspected the stapedius tendon via microscope, providing subjective visual confirmation of the reflex. Both microscope output and audio commentary were recorded.

#### EMG setup

For SM-EMG measurements, either single-use paired monopolar MicroTargeting electrodes (CE 0413, FHC, Romania) or paired disposable steel needle electrodes (TFDN351301, GVB Gelimed, Germany) were used. The EMG measurement system consisted of PowerLab 16/35 and Dual Bio Amp FE135 amplifiers (ADInstruments, Australia), connected to a laptop running LabChart 7 or later (ADInstruments, Australia). Sampling frequency was set to either 10 or 100 kSamples/second and hardware filters were kept to the minimum, allowing a maximum recording spectrum (1–5 kHz).

### Data processing and analysis

#### Online assessment

During surgery, EMG signals were displayed in real time in LabChart using a finite impulse response (FIR) band‑pass filter (100–1000 Hz). The filter had a transition width corresponding to approximately 20% of the cutoff frequency, providing sufficient attenuation of low‑frequency motion artefacts and high‑frequency electrical noise while preserving the EMG bandwidth. For each stimulation sequence, the operators inspected the filtered traces by zooming into the stimulation window to determine whether EMG activity was discernible between the CI‑related stimulation artefacts. To support this assessment, the spectral content of low‑ versus higher‑intensity trials was visually compared to identify increases in components within the EMG bandwidth. These steps allowed the surgical team to give a preliminary intraoperative indication of whether a reflex‑related EMG response was likely present. All intraoperative impressions were subsequently verified, corrected when necessary, and formally classified using the standardized offline post‑processing.

#### Offline assessment

Offline post-processing was performed using MATLAB R2020a or later (The MathWorks Inc, Natick, United States). Processing consisted of four main steps: (1) removal of baseline wondering and power grid noise; (2) zeroing of the stimulation artefact; (3) band-pass filtering (80–800 Hz); (4) EMG envelope and metrics estimation. The initial noise removal was implemented with a conventional high-pass finite impulse response filter with cut-off frequency of 80 Hz. Then, electrical artefacts were identified by detecting samples above 6 times the standard deviation of their vicinity (± 1 s). Then, a second filter discarded spikes that did not match the applied stimulation rate. Based on such mapping, the samples in the artefact region (600 µs) were substituted by zeros. The artefact zeroing was only applied to stimuli with low stimulation rate, for the 2000 pps stimulation, this step was skipped. A band-pass finite impulse response filter was applied to smooth the signal and remove noises outside the 80–800 Hz bandwidth. Finally, the signal envelope was calculated using the root-mean-square (RMS) of the signal in a moving window of 10 ms. RMS of the signal during the stimulation period and prior it was also calculated.

#### Qualitative evaluation

EMG traces were classified as “reflex present” or “no reflex” using a two-step procedure combining quantitative screening and expert visual verification. First, for each stimulation trial, the root-mean-square (RMS) amplitude during the stimulation window was compared with the RMS amplitude prior the stimulation (baseline). Trials showing an RMS ratio ≥ 1.05 (i.e., ≥ 5% increase in activity during stimulation) were flagged as potential reflex responses.

Flagged trials were then reviewed independently by two experts in electrophysiological signals, who assessed whether the waveform exhibited the characteristic pattern of stapedius reflex activation. A valid reflex was defined by (1) physiological morphology, consisting of initial EMG peaks at stimulus onset followed by progressively increasing spike activity throughout the stimulation period; (2) reproducibility across repetitions, such that early peaks had to be present in at least two of the repeated trials at the same intensity; and (3) monotonicity across intensities, meaning that if a clear response was present at a given level, it could not be absent at the next higher level unless the entire response series was deemed unreliable.

Trials were rejected as reflexes when EMG-like activity was judged to be too homogeneous (suggestive of residual stimulation artefact), when isolated noise bursts occurred only within the stimulation window, or when the observed pattern was inconsistent with physiological reflex behaviour. If the independent classifications did not agree, the evaluators discussed the case until consensus was reached; if consensus was not achievable, the most conservative classification (“no reflex”) was applied. A comparison between EMG-based and visually defined eSRT thresholds was done in patients, in which reflexes were detected visually and with EMG using the optimized setup.

For each of these patients, up to twelve CI contacts were tested, and an SR threshold was determined independently for each contact using both EMG and visual inspection. In analogy to acoustically evoked SRs, where thresholds are assessed across multiple frequencies, each CI contact represented one stimulation location and therefore one threshold estimate. Across the five patients, this yielded 26 channel-level threshold pairs in which both methods detected a reflex and the recording quality met the predefined criteria. These channel-level thresholds and their differences were summarized descriptively.

Descriptive statistics were used to report demographic data. Distribution of continuous data was described using mean with standard deviation and median.

## Results

### Patients

**Fig. 1:**
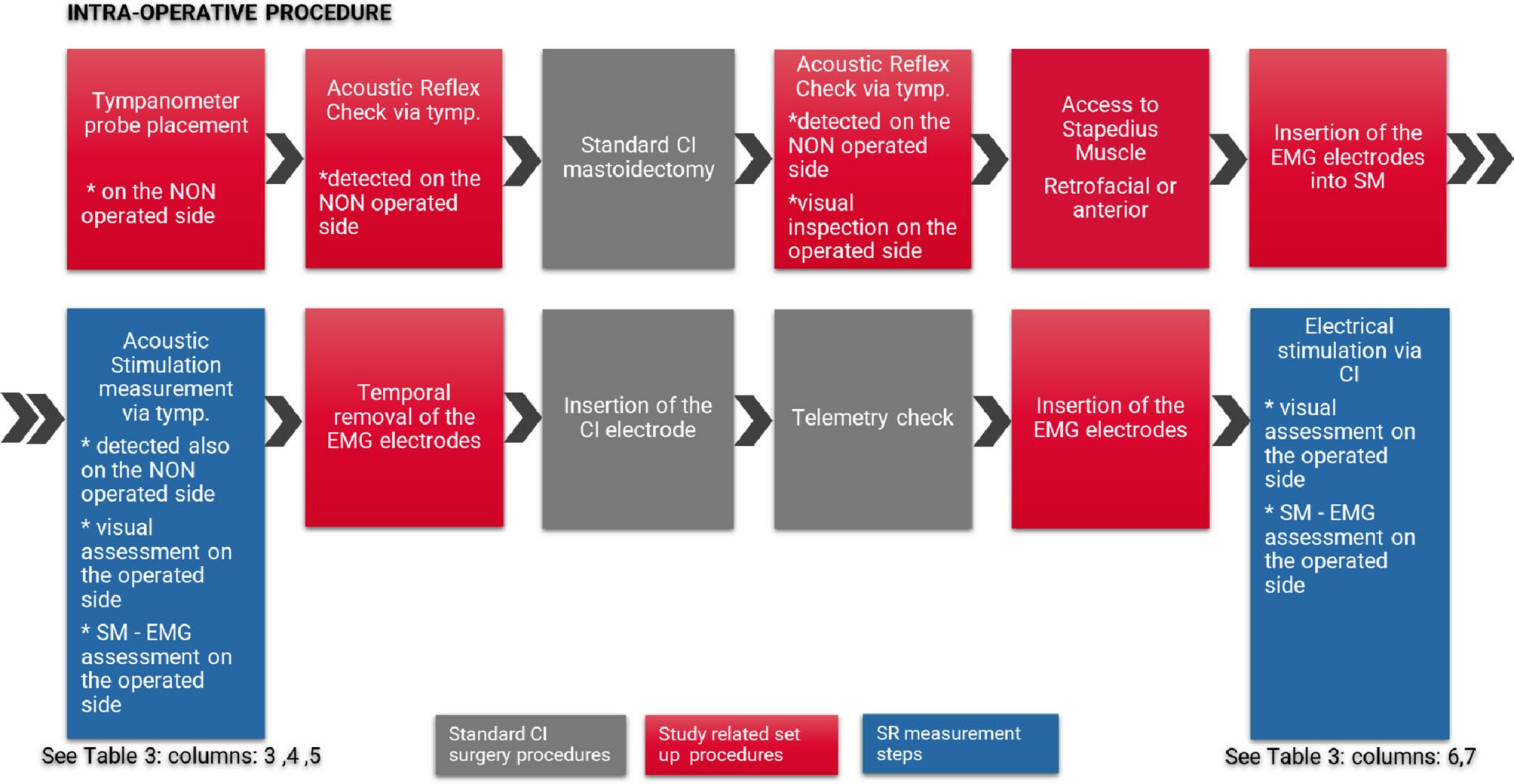
Block diagram summarizing the various phases of the overall process.

Fourteen patients met the inclusion criteria and were included in the study. The CI surgery was scheduled outside of the study. Eight patients were from Jena and 6 patients from Tübingen. The patients’ demographic details are shown in Table 1. Gender and side of implantation was balanced. Median time of single-sided deafness to surgery was 13.1 years. There were no adverse events in any of the patients included for the duration of the study period (Fig. 1).

**Table 1:** Patients’ characteristics.

ID	Age in years	Sex	Side of surgery	Duration of hearing loss at surgery in years
01	30	Male	Right	0.6
02	56	Female	Right	1.3
03	58	Female	Left	35.4
04	57	Female	Left	47.4
05	34	Male	Left	24.5
06	41	Male	Right	1.1
07	57	Male	Left	1.3
08	59	Male	Left	1.1
09	67	Male	Right	63.8
10	40	Female	Left	31.1
11	36	Male	Right	24.3
12	38	Female	Right	1.5
13	57	Male	Left	22.7
14	33	Female	Right	3.6
Mean	47.4	43% Female 57% Male	50% Left 50% Right	18.5
SD	12.4			20.5

### Retrofacial and anterior approach to the stapedius muscle

**Fig. 2:**
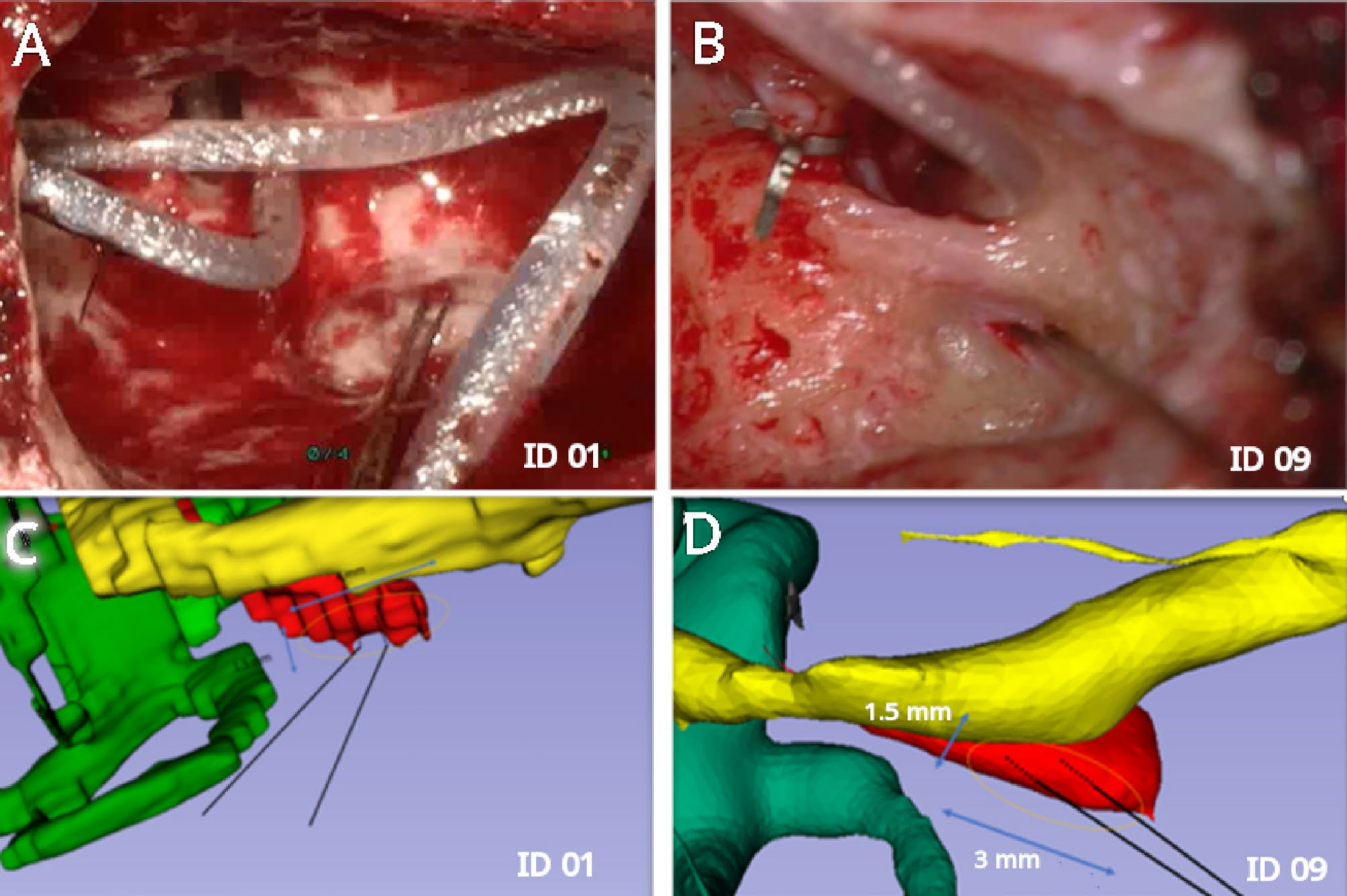
Electrode positioning in the belly of the stapedius muscle using microTargeting electrodes. The real view from the surgical microscope on the upper panels shows the EMG electrodes inserted (highlighted with black lines) from two different patients (**A**: Patient 01 and **B**: Patient 09) and the approximate indication of the drilled window to the surface of the SM belly. The lower panels (**C**: Patient 01 and **D**: Patient 09) show the corresponding 3D reconstructions for the same two patients. The 3D reconstructions were performed with the 3D Slicer software (free download at www.slicer.org; version 5.10.0) as described by Marquez et al. ^17^. The 3D reconstructions are coloured as following: yellow facial nerve; red stapedius muscle, green cochleo-vestibular organ.

Table 2 summarizes the results of the 3D reconstruction for the 14 patients. On average, the 50.04 ± 21.74% of the SM area was exposed posterior-inferior to the mastoid segment of the FN. The distance of the SM to the FN was 1.01 ± 0.22 mm. The depth of the SM posterior to the FN was 1.38 ± 0.81 mm. Table 2 also shows the pre-operative classification (subjective assessment according to the operating surgeon’s visual evaluation) and the corresponding approach performed intra-operatively. Due to the pre-operative evaluation of the 3D reconstructions, it appeared that the SM was concealed in 3 patients, partly exposed in 5 patients, and exposed in 6 patients. Intraoperatively, the belly of the SM was successfully accessed via the retrofacial approach in 11 patients. In 3 patients, a retrofacial approach was not feasible. In such cases, the SM was accessed via an anterior approach. The comparison of the preoperative assessments with the intraoperative situation, showed that when the SM appeared to be exposed in the 3D reconstructions, a retrofacial approach was feasible in all cases. If the SM appeared to be partly exposed, a retrofacial approach was feasible in 11 patients. If the SM appeared from the offset to be concealed, a retrofacial approach was feasible only in 5 patients. Figure 2 shows an example of the optimal trajectories for the insertion of the EMG electrodes in the SM.

**Table 2:** Results of the 3D reconstructions and pre-operative evaluation of the feasibility of a retrofacial approach.

ID	SM exposed area (%)	Distance SM-FN (mm)	Distance SM-SS (mm)	Distance SM-VS (mm)	Depth of SM posterior to FN (mm)	Optimal rotation (degree)	Optimal head tilt (degree)	Diameter of surgical corridor (mm)	Feasible trajectories (%)	Pre-operative evaluation	Approach
01	40.79	1.00	3.94	3.78	0.74	2	0	23.2	0.64	Exposed	Retrofacial
02	29.84	0.55	12.64	2.37	3.02	6	16	2	0.13	Concealed	Anterior
03	25.23	0.89	5.07	5.42	1.08	-2	-8	19.2	0.38	Partly exposed	Retrofacial
04	58.35	1.37	10.13	1.32	2.26	0	-24	26	0.52	Exposed	Retrofacial
05*	N/A	N/A	N/A	N/A	N/A	N/A	N/A	N/A	N/A	Concealed	Retrofacial
06	15.63	0.70	5.60	1.98	1.57	0	-12	3.2	0.11	Partly exposed	Retrofacial
07	45.86	1.08	8.57	3.76	1.55	-2	0	12.4	0.49	Concealed	Anterior
08	60.20	1.07	5.53	2.50	1.63	-4	-14	21.6	N/A	Exposed	Retrofacial
09	57.44	1.19	3.10	2.98	1.33	0	0	13.2	0.77	Exposed	Retrofacial
10	59.44	1.15	5.47	3.31	1.01	2	0	16.4	0.76	Exposed	Retrofacial
11	98.29	0.97	1.19	3.33	0.26	0	0	6.8	0.56	Partly exposed	Retrofacial
12	73.19	1.19	4.60	2.09	0.86	0	0	24.4	0.81	Exposed	Retrofacial
13	36.58	1.08	6.94	4.82	2.36	0	24	9.6	0.37	Partly exposed	Anterior
14	49.65	0.85	2.55	2.03	0.27	-8	-14	8.8	0.29	Partly exposed	Retrofacial
Mean	50.04	1.01	5.79	3.05	1.38	-0.46	-2.46	14.37	0.49	21% Concealed 36% Partly 43% Exposed	21% Anterior 79% Posterior
SD	21.74	0.22	3.15	1.18	0.81	3.28	12.68	8.10	0.24		

SD = standard deviation; N/A = not applicable; SM = stapedius muscle, SS=sigmoid sinus, VS=vestibular system, FN=facial nerve; *data could not be computed because no feasible trajectory was shown i.e., the stapedius muscle was concealed behind the facial nerve.

### Stapedius reflex measurements

**Fig. 3:**
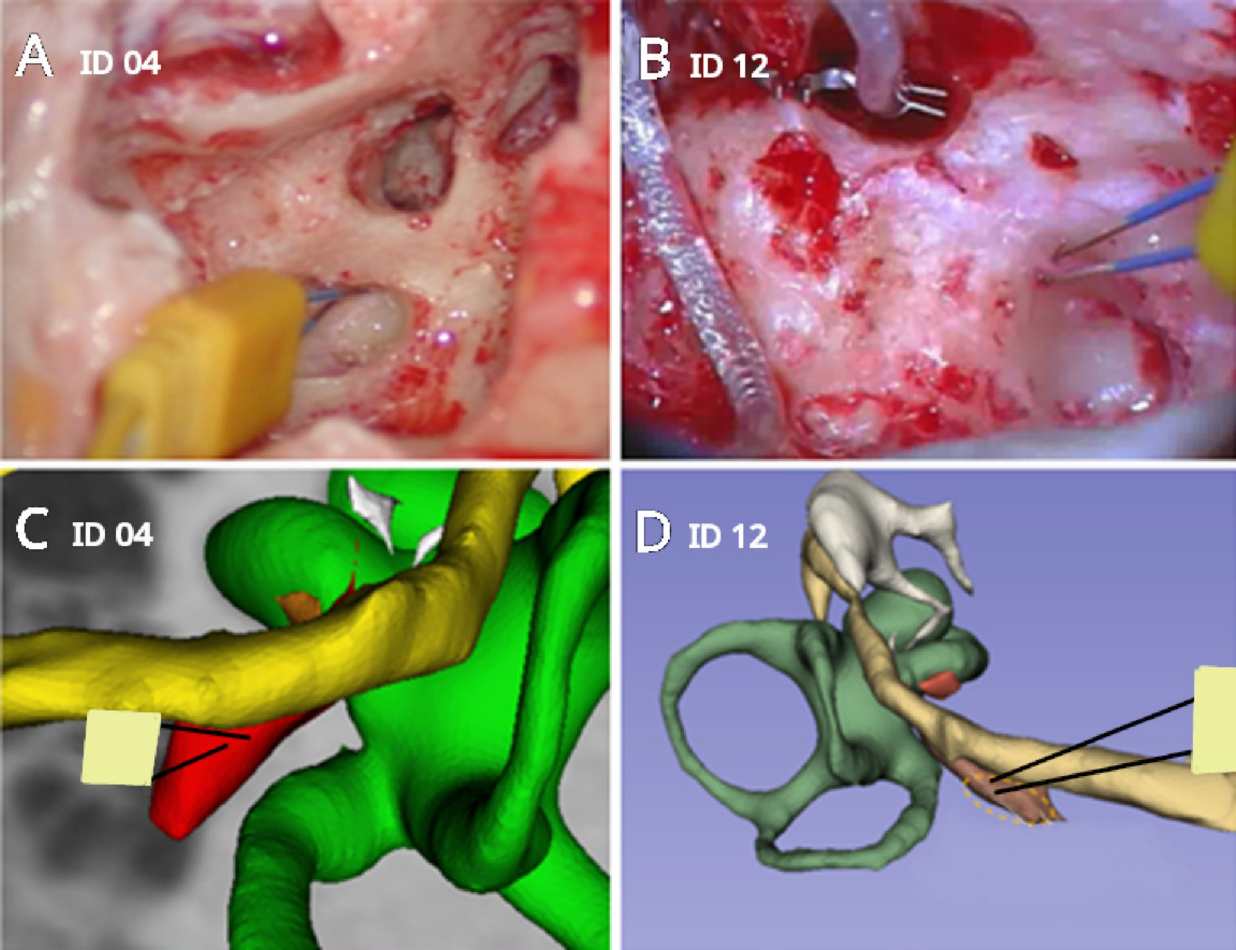
Electrode positioning in the belly of the stapedius muscle via the retrofacial approach using the SpesMedica subdermal pair needles for bipolar electromyography (EMG) recordings. The real view from the surgical microscope on the upper panels shows the EMG electrodes inserted from two different patients (**A**:ID 04 and **B**: ID 12). The lower panels (**C**: ID 04 and **D**: ID 12) show the corresponding 3D reconstructions for the same two patients below each other. The 3D reconstructions were performed with the 3D Slicer software (free download at www.slicer.org; version 5.10.0) as described by Marquez et al. ^17^. The 3D reconstructions are coloured as following: Panel C: yellow facial nerve; red stapedius muscle, green cochleo-vestibular organ. Panel D: yellow facial nerve; brown stapedius muscle, green cochleo-vestibular organ.

Figure 3 shows an example of the paired needle electrodes inserted into the SM via the retrofacial approach, prior and after the insertion of the CI, i.e., during the contralateral acoustic stimulation phase and then during the electrical stimulation via the CI.

**Fig. 4:**
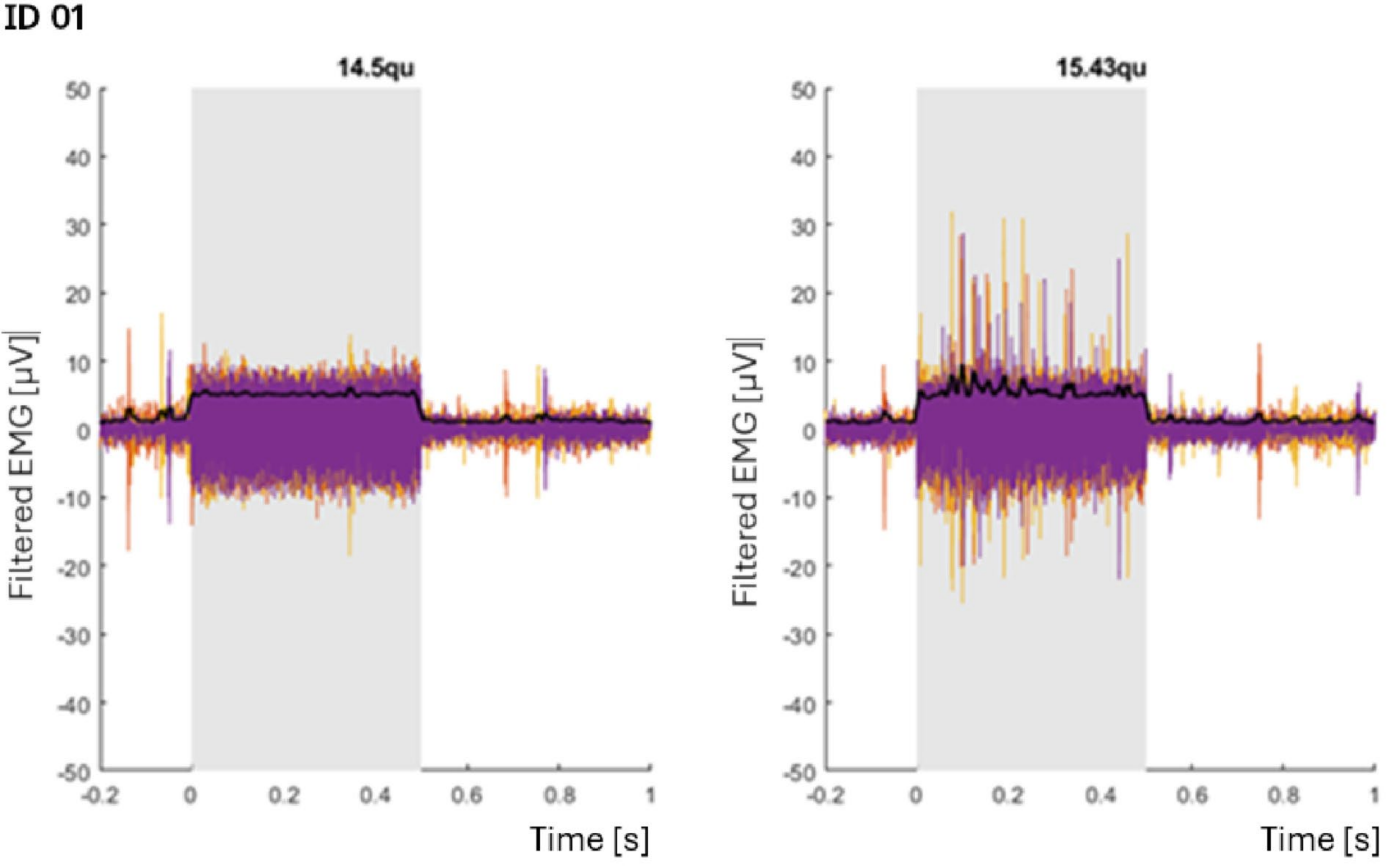
Electromyography (EMG) recordings from one patient (ID 01). The left panel shows three subthreshold stimulations at 14.5 qu, in which only the stimulation artefact is visible. The right panel shows three stimulations at 15.43 qu that elicited clear SM‑EMG activity. In both panels, the black trace represents the averaged EMG envelope across the three individual recordings (orange, purple, and red). The grey shaded region indicates the period during which electrical stimulation was delivered (0–500 ms).

Figure 4 shows an example of EMG recording as performed on the first patient. In the picture two samples of SM-EMG for sub-threshold (left) and supra-threshold (right) stimulations are reported. In the sub-threshold case, only the stimulation artefact is visible for the whole duration of the stimulation (grey area) while in the over- threshold example, the spikes of the EMG signal are visible on top the CI stimulation artefact. Table 3 summarizes the outcome of the acoustic and electric SR stimulation and of the visual or EMG-based confirmations for all the subjects (e.g. yes = at least one successful detection).

**Table 3:** Electromyography (EMG) versus visual assessment of stapedius reflex presence.

ID	Approach	Acoustic stimulation			Electrical stimulation			
		Non-Operated side*	Visual assessment**	EMG assessment	Visual assessment	EMG assessment	Implant / electrode	Contacts stimulated
1	Retrofacial	No	No	Not done	Yes	Yes	SYNCHRONY/ FLEX28	6
2	Anterior	No	No	No	Yes	No	SYNCHRONY/ FLEX28	6, 1, 3
3	Retrofacial	Yes	Yes	No	Yes	No	SYNCHRONY/ FLEX28	2, 6, 9
4	Retrofacial	Yes	No	No	Yes	Yes	SYNCHRONY/ FLEX28	1, 3, 6, 9, 11
5	Retrofacial	Yes	No	Not done	Yes	Yes	SYNCHRONY/ FLEX28	1, 3, 6, 9, 11
6	Retrofacial	Yes	No	Not done	Yes	No	SYNCHRONY/ FLEXsoft	1, 3, 6, 9, 12
7	Retrofacial	Yes	Yes	Yes	Yes	Yes	SYNCHRONY 2/ Standard	1, 3, 6, 9
8	Retrofacial	Yes	No	No	No	No	SYNCHRONY 2/ Standard	1, 3, 6, 9
9	Retrofacial	Yes	No	No	Yes	Yes	SYNCHRONY 2/ Standard	2, 3, 4, 7
10	Anterior	No	No	No	Yes	No	SYNCHRONY 2/ Standard	1, 3, 6, 7, 9, 11
11	Retrofacial	No	Yes	Yes	Yes	No	SYNCHRONY 2/ Standard	1, 5, 11
12	Retrofacial	Yes	Yes	Yes	Yes	Yes	SYNCHRONY 2/ FLEX26	1, 3, 6, 9, 12
13	Anterior	Yes	No	No	Yes	Yes	SYNCHRONY 2/ FLEXsoft	4, 5, 6, 7, 8, 9, 11, 12
14	Retrofacial	Yes	No	Not done	Yes	Yes	SYNCHRONY 2/ FLEX 28	1, 2, 3, 4, 5, 6, 7, 8, 9, 10, 11
Overall	3 anterior 11 retrofacial	10 Yes 4 No	4 Yes 10 No	3 Yes 4 Not done 7 No	13 Yes 1 No	8 Yes 6 No		

* Measured with a tympanometry device on the non-operated side; ** Assessed by visual assessment on the operated side (contralateral to the stimulation). Note: “Yes” indicates that a reflex was detected at least once in at least one stimulation burst.

Visual confirmation of the SR was possible in all but one case (13 patients) for at least 1 frequency (acoustic stimulation) or contact (CI stimulation). The main purpose of recording acoustically evoked SR was to obtain EMG signals that were completely free of stimulation artefacts. Acoustic stimulation was delivered in the non-operated side. An acoustically elicited SR measured intraoperatively in the non-operated side in 10 patients (via tympanometry device, column 3), and in the operated side in 4 patients (via visual assessment, column 4). Of the 4 patients with an acoustically evoked SR on the operated side, it was possible to measure the EMG occurring after the acoustic stimulation in 3 patients (column 5). When electrical stimulation was used, a SM-EMG was detected in 8 out of 14 patients (column 7). In one patient SM-EMG was detected during acoustic stimulation, but not during electrical stimulation; in two cases an electrically evoked SR could be detected, but no acoustically evoked SR.

The study sample was too small to confirm an effect of the approach on the SM-EMG success rate. Electrical stimulation via the CI elicited a SM-EMG in 7 out of the 11 patients with the retrofacial approach, but only in 1 of the 3 patients with the anterior approach.

**Fig. 5:**
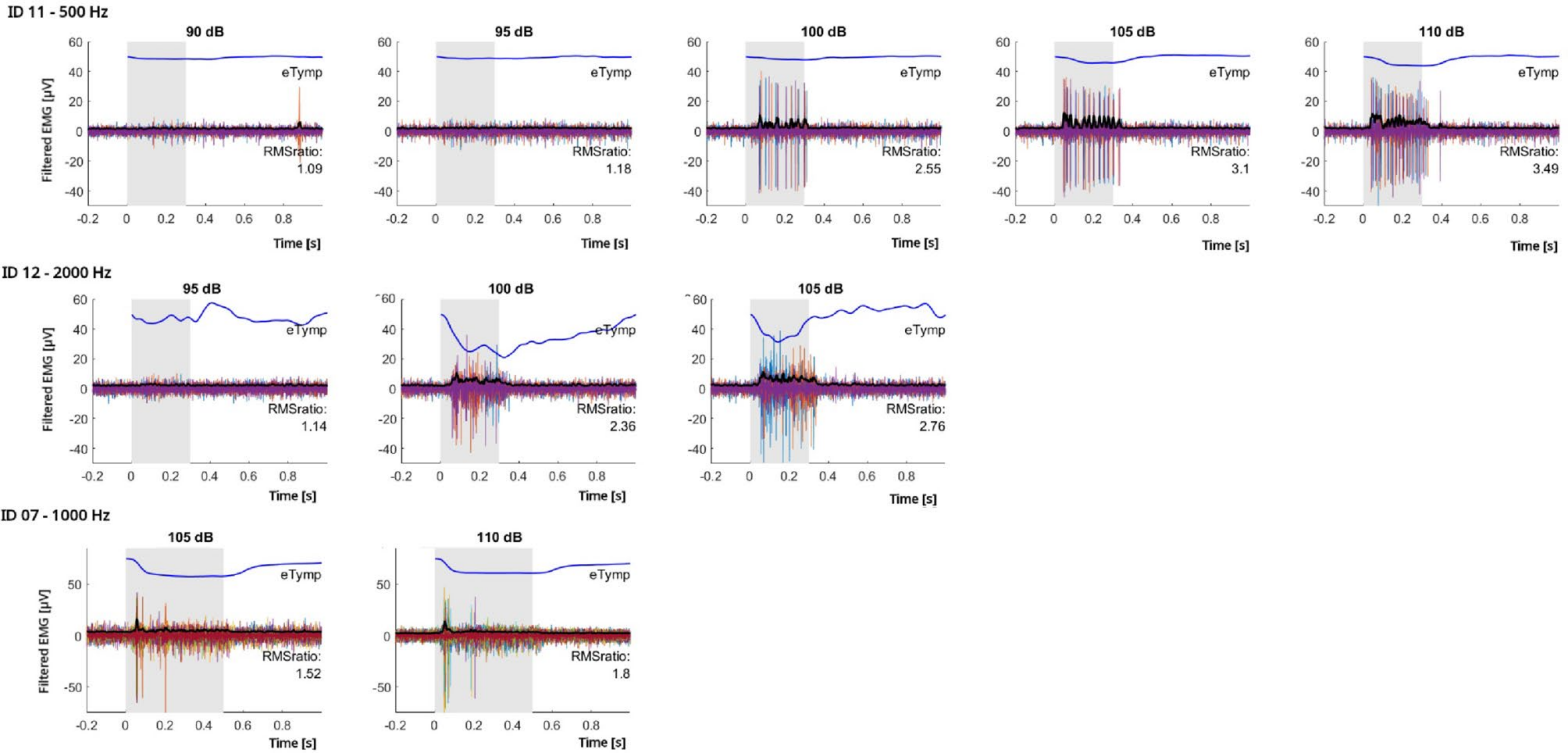
Examples for three different patients of the growing stapedius muscle electromyography activity in response as consequence of contralateral increasing acoustic stimulation. Repeated response for 500 Hz acoustic stimulation at five different intensities is depicted on the top graphs (ID 11). Responses for 2000 Hz acoustic stimulation delivered at3 intensities is represented on the middle graphs (ID 12). Response for 1000 Hz acoustic stimulation at two different intensities is depicted on the bottom graphs (ID 07). In each graph, the blue lines represent the averaged and scaled (factor 300x for visualization reason) tympanometry signal Black lines represent the envelope of the averaged rectified EMG response to repeated stimuli (in the graph reported in the background with yellow, red and purple colours). The grey shaded area represents the interval of time along which the acoustic stimulation was. The tympanometer lines do not refer to the y-axis but are superimposed and synchronized with the stimuli onset. For each graphs x-axis represent the time (s), y-axis represent the amplitude of filtered and rectified EMG signal [µV].

**Fig. 6:**
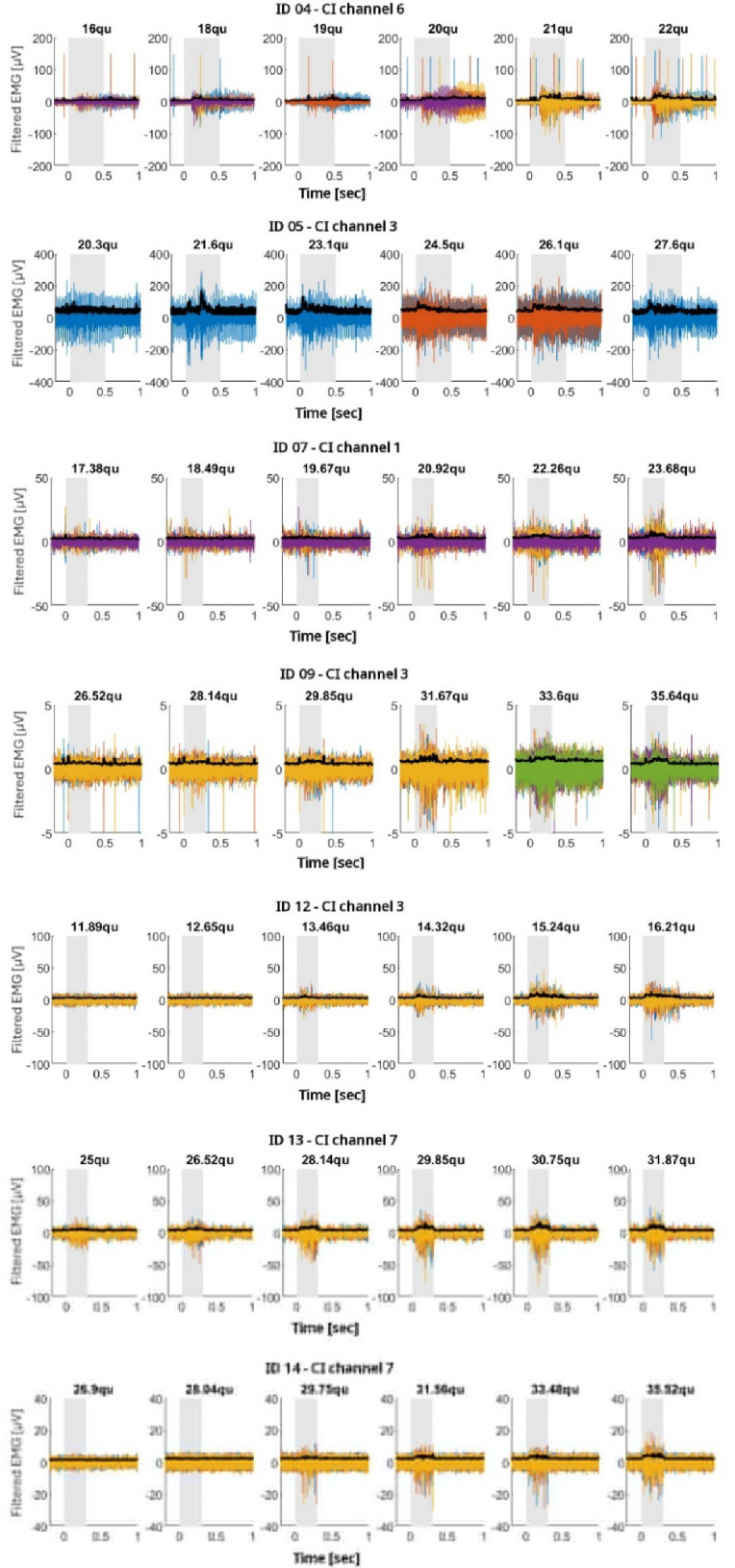
Time course EMG recordings for patients in whom SM-EMG activity was successfully measured after electrical stimulation (see Table 3, column 7). Each row is patient specific and corresponds to one selected CI channel. Each column shows the SM-EMG response elicited by a specific stimulation level (qu) on the indicated channel. The examples illustrate stimulation intensities ranging from sub threshold to supra threshold levels. For all panels, the x axis represents time (sec) and the y axis represents the amplitude of the filtered EMG signal (µV). The black traces represent the averaged EMG envelope across the single individual recordings (overlapped coloured tracks e.g. purple, blue, oranges, yellow, green and red). The grey shaded regions represent the period during which electrical stimulation was delivered. Sub and supra-threshold data for Patient ID 01 have been presented previously in Fig. 4.

Figure 5 shows an example of EMG measurements of the contralateral acoustically evoked SR for three different patients (rows) (ID07, ID11, ID12). The SM-EMG envelope signal represented by the black curves overlies the SM-EMG signal (colored curves). In blue, the averaged and scaled (300x factor for visualization purpose) contralateral acoustically evoked SR measured at different acoustic stimulation intensities (columns) are reported. For the first patient (ID11), a reliable reflex was occurring at 100 dB. As well as for the second patient (ID12). The bottom graphs show the EMG signal in correspondence of very clear and stable SR at high intensities (105 dB and 110 dB) (ID07). Figure 6 shows representative SM EMG traces (from selected CI channels) for all patients in whom electrically evoked SM EMG responses were successfully obtained (see Table 3, column 7). For each patient (each row), SM EMG recordings are displayed across stimulation levels ranging from sub threshold to supra threshold for the selected CI channel. The optimized measurement setup and the refined post processing pipeline (Patients 07, 09, 12, 13, and 14) resulted in cleaner signals compared with those recorded in the first patient group (Patients 04 and 05). Although EMG spikes varied substantially across channels—and even across repeated stimulations at the same channel and intensity—we were able to reliably measure SM EMG responses in more than half of the tested CI channels across multiple stimulation levels.

### EMG versus visual threshold detection

A direct comparison of the threshold values obtained for visual detection versus EMG-based thresholds was done in the five patients where EMG was observed with the refined setup (Patient 07, 09, 12–14). Patients with the initial method were not included as the noise levels might bias the EMG thresholds. This is shown in Fig. 4, where the stimulation artefact completely saturates the signal, and only large EMG signals can be seen when they get higher than the artefacts. A summary of the signal characteristics is shown in Table 4.

**Table 4:** Signal characterization summary.

Parameter	Value
Baseline noise in raw signal peak-to-peak	100–150 µV
Baseline noise in EMG signal peak-to-peak	10–20 µV
EMG amplitude at threshold levels peak-to-peak	20–30 µV
EMG amplitude at high stimulation levels peak-to-peak	80–120 µV
RMS ratio at threshold levels	1.1–1.5
RMS ratio at high stimulation levels	3.5–4.5
CI stimulation artefact in raw signal peak-to-peak	200–600 µV

EMG = electromyography; RMS = root mean square; CI = cochlear implant.

**Fig. 7:**
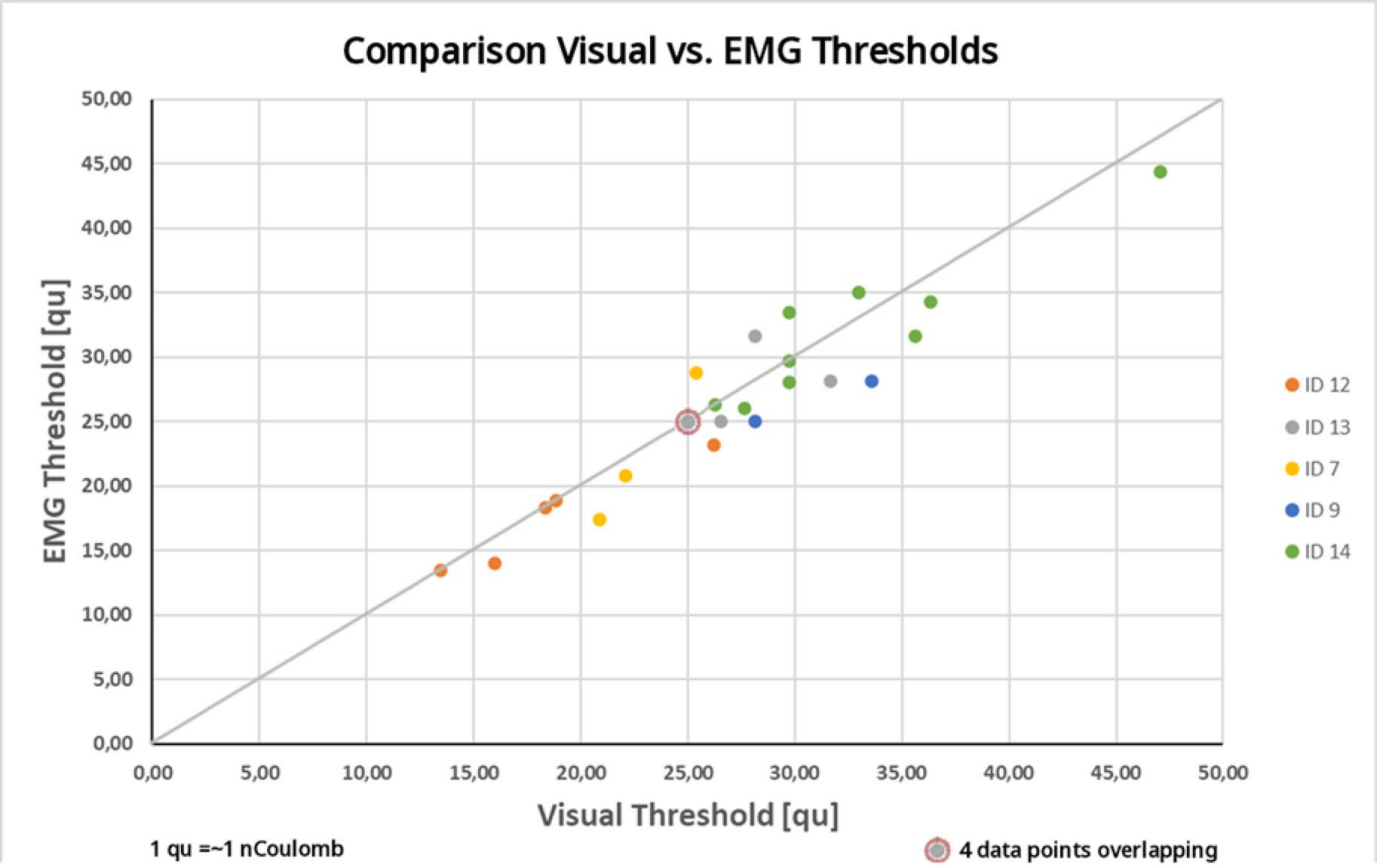
Comparison between the visually-defined stapedius reflex threshold (eSRT) in charge units (qu) and the EMG-defined threshold in qu for different CI channels computed for 5 subjects (Patient 7, 9, 12, 13, 14).

Across the 26 paired channel-level measurements obtained with the optimized setup, EMG-derived thresholds were equal to or lower than visually defined thresholds in 22 cases (85%), including 13 cases (50%) in which the EMG threshold was lower and 9 cases (35%) in which both methods yielded identical thresholds. In only 4 cases (15%) was the visually defined threshold lower than the EMG-derived threshold. The mean reflex threshold (± standard deviation) for visually detection and EMG-based detection was 27.1 ± 7.0 qu and 26.2 ± 6.8 qu respectively. The average paired difference (EMG – visual) was − 0.88 qu, indicating a slight tendency toward lower thresholds when using EMG-based detection. As illustrated in Fig. 7, the distribution of paired differences centered close to zero, suggesting good agreement between methods at the channel level.

**Fig. 8:**
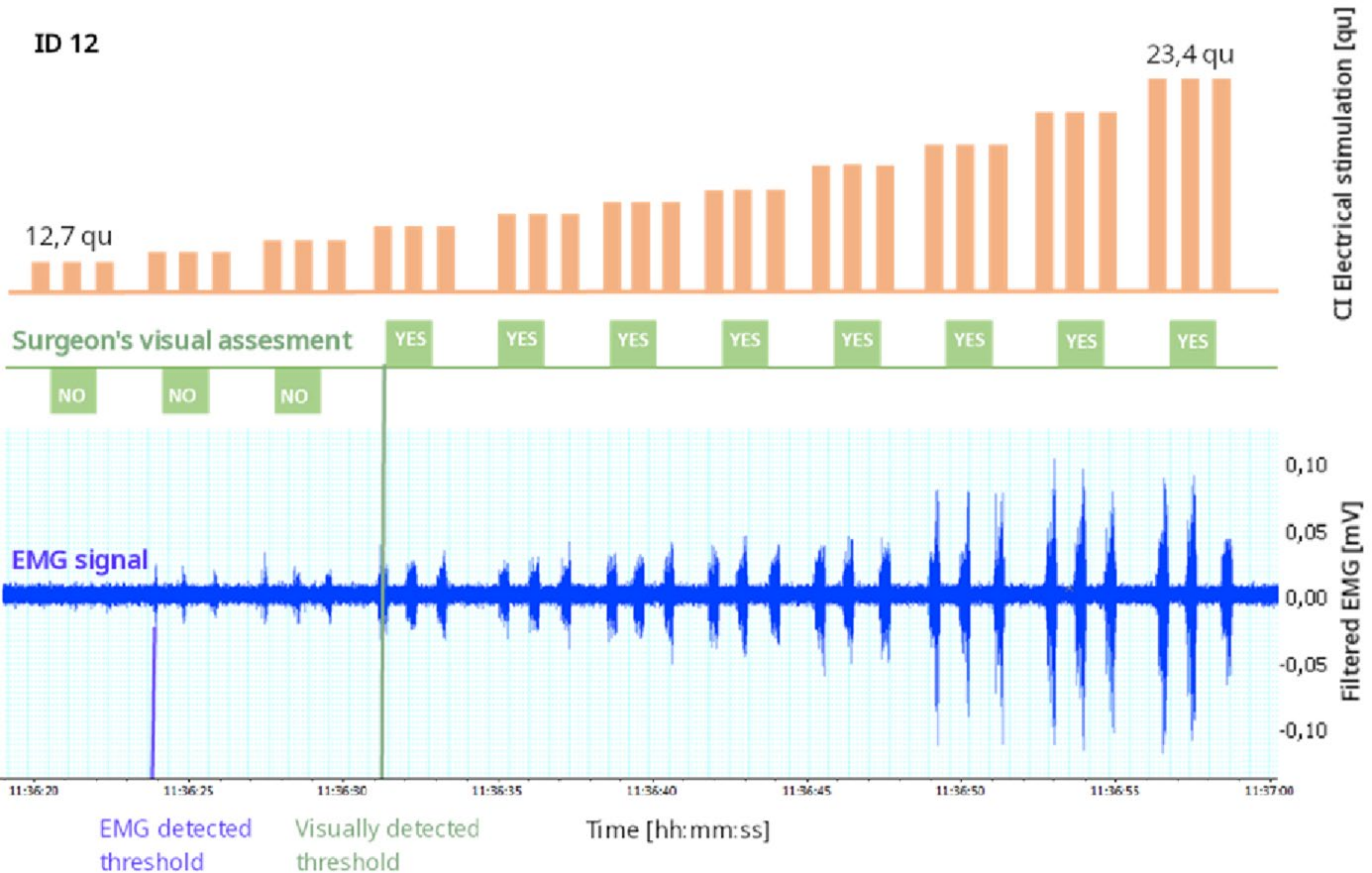
Example of recruitment curve for one patient (Patient 12). The top track in green shows the surgeon’s answer for visual detection of SM movements in correspondence to electrical stimulation delivered via the CI, channel 7. The lower track in blue: shows the SM-EMG signal for the same stimulation. The stimulation ranged from 25 to 45 qu.

Figure 8 shows an example of the elicited EMG responses observed in one patient using different stimulation intensities ranging from low subthreshold (25 qu) to suprathreshold (45 qu); three measurements were performed at each intensity. The results of the visual assessment of the surgeon are also shown for comparison. The comparison shows that, for this case, EMG activity was detected 2 intensity steps before the surgeon detected any movement on the microscope.

## Discussion

The data presented herein demonstrate that objective determination of the SR threshold can be achieved using intraoperative SM-EMG in adults undergoing CI surgery. In the subset of patients with optimized EMG recordings, EMG- and visually defined SR thresholds were strongly correlated and generally similar, indicating that intraoperative SM-EMG can provide threshold estimates that are comparable to the current intraoperative standard based on visual inspection of stapedius tendon movement.

As opposed to subjective behavioral responses, using objective measures such as eSRT for CI fitting does not yet represent the clinical standard despite its usefulness in a clinical setting being proven^18,19^. The measurement of the eSRT during fitting sessions is performed using tympanometry that is able to detect via the external ear canal variation of the admittance in the middle ear occurring during the SR; thus, providing an indirect measure of SM activity^12^. However, in cases with middle-ear malformations or conditions where the biomechanics are not intact tympanometry cannot be used^13,20^. However, using intraoperative approaches that measure the electromyographic signal direct from the EMG source (muscle belly), SM activity can also be determined in cases of malformed anatomy (e.g. missing SM tendon).

The main challenge for the intraoperative eSRT measurement via retrofacial approach is the accessibility to the SM, which is encased by bone with only a narrow opening for the tendon that connects it to the stapes. Ideally, the electrodes should be inserted parallel to the SM length so that a larger area of muscle fibers can be reached. In practice, however, this was difficult as the anatomy varied and the access to the SM did not allow such a flexibility in the direction of the insertion of the electrode in all the cases. In the present study, in most cases (11 out of 14 patients) the retrofacial approach could be used to successfully access the SM. Thus, the study confirmed the finding of our cadaver in which we determined that the retrofacial approach was feasible for accessing the SM^14^. The feasibility of the retrofacial approach for cochlear implantation was investigated by Yilmazer et al.^21^, before we documented using the approach to access the stapedius muscle in 2022 during CI implantation^15^. The SM can be accessed directly and safely using the approach particularly when the SM was categorized as ‘exposed’ with respect to the facial nerve.

Our results showed that it is possible to reliably measure SM-EMG signal directly from the SM muscle belly and that the higher is the electric/ acoustic stimulation eliciting it, the higher is the number of recruited motor units, the stronger is the signal which is measured. One main limitation of our measurements - when reflex is elicited via CI - is the effects of the CI stimulation itself. Due to the location of the SM close the where the CI is implanted, the EMG signal is contaminated by electrical artefact noise coming from the CI stimulation. Therefore, post processing of the signal is a necessary step. In addition to filtering the CI artefact, we obtained contralateral acoustically elicited SM-EMG recordings. These measurements were acquired without any CI stimulation and therefore without CI-related artefacts. Their purpose was solely to collect evidence of the morphology of a physiologically driven SM-EMG signal, providing insights supporting the interpretation that the EMG activity observed during CI stimulation corresponds to genuine SM-EMG.

Intraoperative direct measure of the SM-EMG has previously been documented^22–24^. Using a different form to our approach the SM-EMG was determined by accessing the SM pyramidal eminence. However, although such an approach reduces the proximity to anatomical structures such as the facial nerve, it faces other drawbacks such as possible damage to the stapedius tendon and there is a lower number of the muscle fibers in such a location, therefore lower amplitudes are expected in the SM-EMG signal.

A secondary aim of this study was to compare intraoperative SR thresholds obtained via SM-EMG with those derived from the surgeon’s visual inspection of stapedius tendon movement. The comparison was restricted to the five patients measured with the optimized configuration because, in the initial recordings, noise levels were too high to reliably observe EMG responses near threshold.

Visual assessment is inherently subjective^25^, and although video-based tracking tools have been proposed to improve precision^26^, these systems are not widely used clinically and were not implemented here, as our comparison served only to verify the technical plausibility of the EMG approach rather than to benchmark clinical performance.

Because EMG detects the earliest recruitment of motor units—before force generation produces a visible tendon movement —EMG thresholds are expected to occur at the same or lower stimulation levels than visual thresholds. Accordingly, in most of the analyzable channel-level pairs obtained from the five patients recorded with the optimized setup, EMG thresholds were equal to or lower than visual thresholds. Although the average difference between visually and EMG‑determined thresholds was small and no statistical conclusions can be drawn from it, the pattern observed is compatible with the physiological expectation that EMG may register early motor‑unit activation before a visible stapedius contraction occurs. When stimulation increments are relatively large, however, EMG and visually defined thresholds may coincide, as happened in nine cases. In the remaining cases where the visually determined threshold appeared lower, the most likely explanations are technical: insufficient signal-to-noise ratio due to intraoperative noise, partial removal of low amplitude EMG during artefact suppression, or suboptimal electrode placement limiting contact with the SM belly. Optimizing these factors is therefore essential to maximize the reliability of EMG based detection.

Overall, the comparison supports the expected physiological relationship between EMG based and visually defined SR detection and confirms the technical validity of the EMG approach, while highlighting areas where further optimization—particularly noise reduction, artefact handling, and electrode positioning—is needed.

Furthermore, anesthesia may have an effect on the eSRT measurement. Intraoperative eSRT measurements occasionally show different results to post-operative. The differences are thought to be due to the use of muscle relaxants and anesthesia in the intra-operative setting^25,27^. Depending on the type and the dose of anesthesia the eSRT response can be incremented, diminished, or even eliminated^28–31^. However, the effect of anesthesia was not determined on the protocol herein.

Several limitations of the study have to be addressed. The present study was also limited by the age group as some auditory responses are typical in certain age groups, thus the lack of data across all age groups limits some inferences. In particular as the procedure used during the mapping of CIs differs in regard to the CI recipient’s age and also the duration of hearing deprivation prior to activation of the CI^32–34^. Thus, the approach would also need to be verified in children, as only adults were analyzed herein. Only patients with single-sided deafness and preserved contralateral acoustically evoked SR were included. Next step, we also have to confirm the feasibly also for patients with bilateral severe hearing loss or deafness. Also, the sample size of our study was relatively small. Nonetheless, the intention was a proof of principle clinical investigation, and this has been successfully achieved.

The placement of the electrode for signal quality and the type of electrode may also have an effect. A large difference was found in the success rate of EMG measurements in patients where the retrofacial approach was used (11 patients) compared to those where the anterior approach was the only option (3 patients). This may be partially due to the higher density of tendinous tissue in the access area, but also to the usage of bipolar needles with a large surface area, which were selected primarily for the optimization of measurements from the SM belly and not from the tendinous segment. As shown elsewhere^24^, an anterior approach can also be optimized by using certain type of electrodes, even with a monopolar configuration and has the advantage of a simpler surgical approach and fixation. On the other hand, special attention must always be given to the electrical artefact on the SM-EMG signal produced during CI stimulation because, as shown in this study, it can be a limiting factor for the success and sensitivity of the measurement of eSRTs.

In its actual form, the intraoperative retrofacial or anterior access and the temporary EMG electrodes should be regarded primarily as a research tool for validating SM‑EMG recordings during CI stimulation. The intention is not to replace established intraoperative objective measures such as eCAP or eABR, but rather to provide a foundation for future CI systems capable of chronic SM‑EMG monitoring and potential closed‑loop applications.

## Conclusions

The first clinical study in 14 patients undergoing CI surgery for single-sided deafness showed the feasibility of detecting the SR via EMG measurements directly from the belly of the SM. From the surgical point of view, the retrofacial approach was best suited for patients with an exposed or partially exposed SM respect to the facial nerve, with the best trajectory is in patients with an exposed SM. Therefore, pre-operative planning using high-resolution CT data was mandatory. In general, the procedure was safe, reproducible and reversible. Further investigation is needed to study the relationship between intraoperative SM-EMG-based eSRT and patient’s current levels, to determine how it may facilitate CI programming. Then, it could represent a concrete step towards future closed-loop smart CI devices for measurement during CI fitting.

## Data Availability

Data is provided within the manuscript. All authors had full access to all of the data in the study. O.G.-L. takes responsibility for the integrity of the data and the accuracy of the data analysis. O.G.-L. should be contacted if someone wants to request the data from this study.

## References

[1] Deafness and hearing loss. Vol. (World Health Organization, (2024). https://www.who.int/news-room/fact-sheets/detail/deafness-and-hearing-loss, 2024).

[2] Vaerenberg, B. et al. Cochlear implant programming: a global survey on the state of the art. *Sci. World J.* **1**, 501738 (2014). 10.1155/2014/501738 PMC3932199 24688394

[3] Psarros, C. & Abrahams, Y. Recent Trends in Cochlear Implant Programming and (Re)habilitation. in Cochlear Implants: New and Future Directions (Sandra DeSaSouza ed). 441–471 (Springer Nature Singapore, Singapore, (2022).

[4] de Andrade, K. C. L. et al. The Value of Electrically Evoked Stapedius Reflex in Determining the Maximum Comfort Level of a Cochlear Implant. *J. Am. Acad. Audiol.* **29**, 292–299 (2018). 29664723 10.3766/jaaa.16117

[5] Lindström, B. & Bredberg, G. Intraoperative electrical stimulation of the stapedius reflex in children. *Am. J. Otol*. **18**, S118–119 (1997). 9391626

[6] Spivak, L. G. & Chute, P. M. The relationship between electrical acoustic reflex thresholds and behavioral comfort levels in children and adult cochlear implant patients. *Ear Hear.* **15**, 184–192 (1994). 8020651 10.1097/00003446-199404000-00008

[7] Bresnihan, M., Norman, G., Scott, F. & Viani, L. Measurement of comfort levels by means of electrical stapedial reflex in children. *Arch. Otolaryngol. Head Neck Surg.* **127**, 963–966 (2001). 11493206 10.1001/archotol.127.8.963

[8] Gordon, K. A., Papsin, B. C. & Harrison, R. V. Toward a battery of behavioral and objective measures to achieve optimal cochlear implant stimulation levels in children. *Ear Hear.* **25**, 447–463 (2004). 15599192 10.1097/01.aud.0000146178.84065.b3

[9] Kosaner, J., Anderson, I., Turan, Z. & Deibl, M. The use of ESRT in fitting children with cochlear implants. *J. Int. Adv. Otology*. **5**, 70–79 (2009).

[10] Jerger, J., Oliver, T. A. & Chmiel, R. A. Prediction of dynamic range from stapedius reflex in cochlear implant patients. *Ear Hear.* **9**, 4–8 (1988). 3342943 10.1097/00003446-198802000-00010

[11] Stephan, K. & Welzl-Müller, K. Stapedius reflex in patients with an inner ear prosthesis. *Int. J. Artif. Organs*. **15**, 436–439 (1992). 1516996

[12] Schairer, K. S., Feeney, M. P. & Sanford, C. A. Acoustic reflex measurement. *Ear Hear.* **34** (Suppl 1), 43s–47s (2013). 23900179 10.1097/AUD.0b013e31829c70d9

[13] Kopuz, C., Turgut, S., Kale, A. & Aydin, M. E. Absence of both stapedius tendon and muscle. *Neurosciences (Riyadh)*. **11**, 112–114 (2006). 22266561

[14] Arnold, D. et al. Accessing the Stapedius Muscle Via Novel Surgical Retrofacial Approach: A Cadaveric Feasibility Study. *Otol Neurotol*. **43**, e174–e180 (2022). 34855681 10.1097/MAO.0000000000003413

[15] Guntinas-Lichius, O. et al. Accessing the stapedius muscle via novel surgical retrofacial approach during cochlear implantation surgery: Intraoperative results on feasibility and safety. *PLoS One*. **17**, e0272943 (2022). 35951500 10.1371/journal.pone.0272943 PMC9371293

[16] Volk, G. F. et al. Dyna-CT of the temporal bone for case-specific three-dimensional rendering of the stapedial muscle for planning of electrically evoked stapedius reflex threshold determination during cochlear implantation directly from the stapedius muscle via a retrofacial approach: a pilot study. *Eur. Arch. Otorhinolaryngol.* **277**, 975–985 (2020). 31897721 10.1007/s00405-019-05773-2

[17] Marquez, P. et al. The use of a surgical planning tool for evaluating the optimal surgical accessibility to the stapedius muscle via a retrofacial approach during cochlear implant surgery: a feasibility study. *Int. J. Comput. Assist. Radiol. Surg.* **16**, 331–343 (2021). 33185757 10.1007/s11548-020-02288-8 PMC7880982

[18] Raghunandhan, S., Ravikumar, A., Kameswaran, M., Mandke, K. & Ranjith, R. A clinical study of electrophysiological correlates of behavioural comfort levels in cochlear implantees. *Cochlear Implants Int.* **15**, 145–160 (2014). 24606544 10.1179/1754762814Y.0000000064

[19] Mishra, R. & Nandurkar, A. Correlation between behaviorally measured comfort (c) levels and electrically evoked stapedius reflex thresholds (ESRT) in children with unilateral cochlear implant. *Journal Otolaryngology-ENT Research* **11**, 201-6 (2019).

[20] Djerić, D. & Savić, D. [Anatomical variations and anomalies of the musculus stapedius tendon. Study by scanning electron microscopy]. *Ann. Otolaryngol. Chir. Cervicofac.* **104**, 59–63 (1987). 3566051

[21] Yilmazer, R. et al. The Feasibility of Retrofacial Approach for Cochlear Implantation. *Otol Neurotol*. **39**, e550–e556 (2018). 29957670 10.1097/MAO.0000000000001878

[22] Pau, H. W. et al. Electromyographical recording of the electrically elicited stapedius reflex via a bipolar hook electrode. *Otol Neurotol*. **30**, 1–6 (2009). 18833019 10.1097/MAO.0b013e31818a0898

[23] Almqvist, B., Harris, S. & Shallop, J. K. Objective intraoperative method to record averaged electromyographic stapedius muscle reflexes in cochlear implant patients. *Audiology* **39**, 146–152 (2000). 10905400

[24] Zarowski, A. et al. Intraoperative recordings of electromyogenic responses from the human stapedius muscle. *Hear. Res.* **408**, 108290 (2021). 34233241 10.1016/j.heares.2021.108290

[25] Pau, H. W., Ehrt, K., Just, T., Sievert, U. & Dahl, R. How reliable is visual assessment of the electrically elicited stapedius reflex threshold during cochlear implant surgery, compared with tympanometry? *J. Laryngol Otol*. **125**, 271–273 (2011). 21054912 10.1017/S0022215110002392

[26] Weiss, B. G. et al., An Objective Method to Determine the Electrically Evoked Stapedius Reflex Threshold During Cochlea Implantation.* Otology & Neurotology* **39**, e5-e11 (2018). 10.1097/MAO.0000000000001611 29116963

[27] Van Den Abbeele, T. et al. Multicentre investigation on electrically evoked compound action potential and stapedius reflex: how do these objective measures relate to implant programming parameters? *Cochlear Implants Int.* **13**, 26–34 (2012). 22340749 10.1179/1754762810Y.0000000001

[28] Gnadeberg, D. et al. [Effect of anesthesia on the intraoperative elicited stapedius reflex]. *Laryngorhinootologie* **73**, 132–135 (1994). 8172632 10.1055/s-2007-997095

[29] Makhdoum, M. J., Snik, A. F., Stollman, M. H., de Grood, P. M. & van den Broek, P. The influence of the concentration of volatile anesthetics on the stapedius reflex determined intraoperatively during cochlear implantation in children. *Am. J. Otol*. **19**, 598–603 (1998). 9752967

[30] Schultz, A. et al. Intraoperative electrically elicited stapedius reflex threshold is related to the dosage of hypnotic drugs in general anesthesia. *Ann. Otol Rhinol Laryngol*. **112**, 1050–1055 (2003). 14703109 10.1177/000348940311201210

[31] Crawford, M. W. et al. Dose-dependent suppression of the electrically elicited stapedius reflex by general anesthetics in children undergoing cochlear implant surgery. *Anesth. Analg*. **108**, 1480–1487 (2009). 19372325 10.1213/ane.0b013e31819bdfd5

[32] Andrade, K. C. et al. The importance of electrically evoked stapedial reflex in cochlear implant. *Braz J. Otorhinolaryngol.* **80**, 68–77 (2014). 24626895 10.5935/1808-8694.20140014 PMC9443964

[33] Yathiraj, A., Manjula, P., Geetha, C., Jawahar Antony, P. & Megha Comparison of electrically evoked stapedial reflexes in patients with cochlear implants surgically implanted using Veria and posterior tympanotomy approaches. *The J. Laryngology & Otology*, **138**, 858–863 (2024). 10.1017/S0022215124000227 38311334

[34] Hodges, A., Butts, S. & King, J. *Cochlear Implants Objective Measures* 81–96 (Whurr publishers Los Angeles, 2003).

